# Remineralization ability of two hydraulic calcium-silicate based dental pulp capping materials: Cell-independent model

**DOI:** 10.4317/jced.55689

**Published:** 2019-04-01

**Authors:** Salma M. Fathy

**Affiliations:** 1 Dental Biomaterials Dept., Faculty of Oral and Dental Medicine, Zagazig University, Egypt

## Abstract

**Background:**

This study aimed to evaluate remineralizing ability of two hydraulic calcium-silicate cements (Biodentine and TheraCal LC).

**Material and Methods:**

Artificial carious lesions were introduced into the pulpal floors (1-1.5 mm) and axial walls of occlusal prepared cavity halves through pH cycling. Cycling was made through demineralizing solution (pH 3), for 8 hours and remineralizing solution (pH 7) for 16 hours. The total period of pH cycling was 14 days. Prepared cavities with the tested materials seated directly on the pulpal floor and in contact with the axial walls were stored in phosphate buffer solutions (PBS) (pH 7.2–7.4). The changes in the weight percentages (wt%) of calcium (Ca) and phosphorus (P) were detected using SEM and energy dispersive X-ray spectroscopy with reference to sound dentin after three intervals (one week, 3 and 6 months). Data were statistically analyzed using two-way ANOVA and Tukey’s post hoc test.

**Results:**

Demineralized dentin, next to Biodentine, showed statistically higher intensities of Ca and P wt% after the three periods of incubation (*p*< 0.05). Surface mapping of both tested cements and their adjacent demineralized dentin showed increase in overall distribution of previous ions. SEM of subsurface layer under both materials showed filling of most intra-tubular areas with rod-like mineralized structure without significant difference.

**Conclusions:**

Biodentine has a higher ability to enrich the artificial carious dentin with significantly higher mineral contents available for remineralization. Both pulp-capping materials have significantly induced remineralization of demineralized dentin beneath them after total period of incubation.

** Key words:**Artificial Caries, Hydraulic Cements, pH Cycling, Remineralization.

## Introduction

The modern approach of caries treatment involves removal of only the infected carious dentin, leaving the internal caries affected dentin that can be remineralized (1). As a result, it is crucial to evaluate the ability of recently introduced capping materials to remineralize and regenerate dentin (2). Proper interaction of hydraulic calcium-silicate cements (HCSCs) with the remaining caries affected dentine, in the presence of healthy diagnosed pulp, allows biological remineralization response of the carious lesion (3).

HCSCs have proven their ability to serve as permanent dentin substitutes mainly due to their antibacterial effect and associated re-mineralization ability. Biodentine was reported to have clinically acceptable setting times and physical properties especially in comparison to mineral trioxide aggregate (MTA) (4,5). A number of recent studies demonstrated that HCSCs, including Biodentine (Septodont, St. Maurdes Fossis, France) and TheraCal LC (Bisco Inc., Schamburg, IL, USA), possess bioactive properties when immersed in phosphate-based solutions such as simulated body fluid (2,6). They are able to induce the formation of apatite precipitates. However, the stability of the interfacial mineralized area between Biodentine and tooth dentin was questionable. Only amorphous Ca phosphate was identified not dentin like hydroxyapatite within the former area (7).

The polymer matrix of light curable tricalcium silicate-based material (TheraCal LC) was mentioned in its patent claims as being a hydrophobic monomer or combination of monomers such as bis-phenyl glycidyl methacrylate (Bis-GMA) and other acrylates in combination with at least one hydrophilic monomer like 2-hydroxyethylmethacrylate (2-HEMA) (8). The presence of a hydrophilic resin, within TheraCal LC, is essential for hydration of HCSCs and calcium release for apatite formation (2). However, a high degree of polymerization for the resin matrix of this composite HCSC may induce a negative effect on the sustained release of Ca ions and thus the long-term remineralization. The current study aims to evaluate remineralization ability of resin-free HCSC (Biodentine) and resin-based HCSC (TheraCal LC), in the presence of artificial carious dentin barrier (cell-independent model) simulating the condition of indirect pulp capping.

## Material and Methods

Two types of HCSCs were used in the present study. The first is a calcium-silicate based powder mixed with hydrosoluble polymer in liquid (Biodentine). The other is a resin composite-calcium silicate based cement (TheraCal LC) ( Table 1).

**Table 1:**
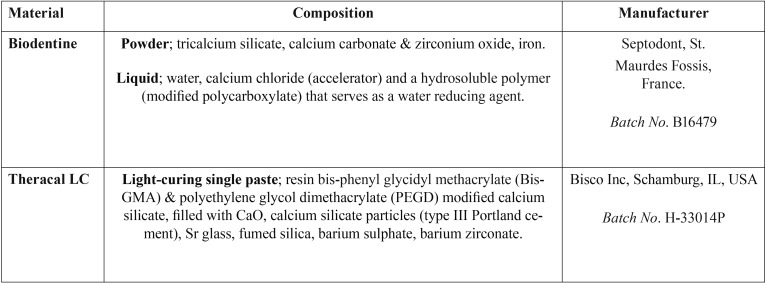
Materials used in the present study.

-Cell-independent model for testing remineralization 

1. Cavity design

**Figure 1:**
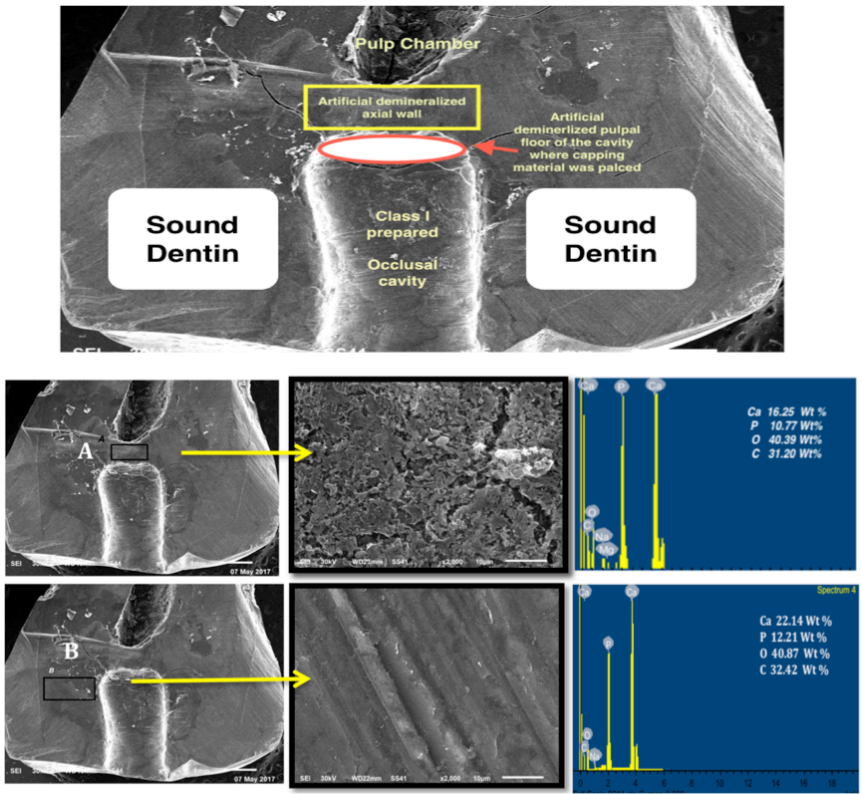
Low magnification SEM images (x75) illustrating, the design of the occlusal prepared cavity, selected areas of induced dentine demineralization (A), protected sound dentine (B) and the position of tested materials (top image). The yellow arrows are pointing to high magnification SEM images (x2000) of A and B areas with EDS analysis of their surfaces showing porous rough surface with lower Ca & P Wt% for A than B.

Forty healthy human maxillary premolars were collected from the Faculty of Dentistry external clinic’s patients (under the approval of Medical Ethics Comity No. 1480518). Extracted teeth were examined using an USB digital light microscope (Scope Capture, Guangdong, China) connected with an IBM compatible personal computer at x10 magnification. Teeth with caries or enamel cracks were discarded. The remaining teeth were ultrasonically cleaned, then stored in 0.5% thymol solution at 4oC until the time of use. The roots of all teeth were removed at the cemento-enamel junction. The crowns were then sectioned vertically in a mesio-distal direction into two buccal and lingual halves, using a diamond microsaw (IsoMet 400, Buehler, Lake Bluff, Illinois, USA) at 2500 rpm speed and continuous water-cooling. The pulp tissue in each crown half was removed using a spoon excavator. A deep occlusal cavity preparation was done toward the pulpal roof using a medium-grit (107 μm) diamond bur (842, Komet, Lemgo, Germany) fixed in a water-cooling high-speed turbine. The prepared cavity width was 1.8-2.2 mm in mesio-distal dimensions. Pulpal floor thickness was 1-1.5 mm of remaining dentin measured from central portion of pulpal floor toward the roof of pulp chamber. This thickness lies within the range previously used for dentin demineralization approaches (9,10). The entire half crown was covered with an acid-resistant acrylic varnish (Ultimate Shine, Top Coat, Revlon, New York, USA) except for the pulpal floor and axial wall directly above the pulp chamber. These former and later areas were protected using celluloid tape (Fig. 1).

2. Artificial caries induction and tested materials placement

The induction of artificial carious lesion in dentin was produced using a pH cycling protocol (11). The demineralizing period was 8 hours and remineralizing was 16 hours. The composition of both remineralizing solution (pH 7) and demineralizing solution (pH 3) was previously described (11). The specimen size of the tested HCSCs to be placed on the cavity floor was standardized using a split Teflon mold 5 mm diameter and 1 mm thickness. This volume of was then placed, condensed, and made flush within the floor and walls of the prepared cavity (Fig. 1). Condensation of the material was done using a plastic cement spatula and manipulation was done according to the manufacturer’s instruction. A negative control group of 8 empty artificial carious cavities for each time interval were also evaluated. All groups were immersed in phosphate buffered solutions (PBS of pH 7.2–7.4) with composition of 1.6 g NaCl, 0.04 g KCl, 0.288 g Na2HPO4, and 0.048 g KH2PO4 for each 200 ml (12). The specimens were stored at 37oC comparable to patient mouth temperature. Sample groups were examined using scanning electron microscope (SEM), energy dispersive x-ray spectroscopy (EDS), and surface mapping (SEM, Quanta FEG 250, FEI, Eindhoven, Netherlands) at intervals of one week, and 3 and 6 months (6). The mineral weight percentage (wt%) of calcium (Ca) and phosphorus (P) within demineralized dentin (DMD), adjacent to the capping material, and within the protected sound dentin (SD) were evaluated. The mineral percentage difference between DMD and SD in each tooth half was calculated. After evaluation of the SD surface mineral content of each tooth half using EDS, it was taken as the specimen reference (i.e., 100% mineral content of such specimen). The DMD mineral content of each specimen was then calculated in relation to this reference value for the different intervals of storage. Afterwards, the capping materials were removed from selected specimens. Their pulpal cavity floors were abraded with successive abrasives papers to #4,000 grit to remove a few microns from surface layer. Subsurface composition and structure were examined using SEM and EDS.

-Statistical analysis

Statistical analysis was performed using SPSS 16.0 (SPSS, Chicago, IL, USA) for Windows. The wt% of Ca and P at different intervals were analyzed using two-way ANOVA test. The normal distribution of the data was first assessed by means of Shapiro and Wilk test. Tukey’s post hoc test was used afterwards. Significance was determined at *p* < 0.05. Results were expressed as mean ± standard deviation (SD).

## Results

-Cell-independent remineralization ability

**Figure 2:**
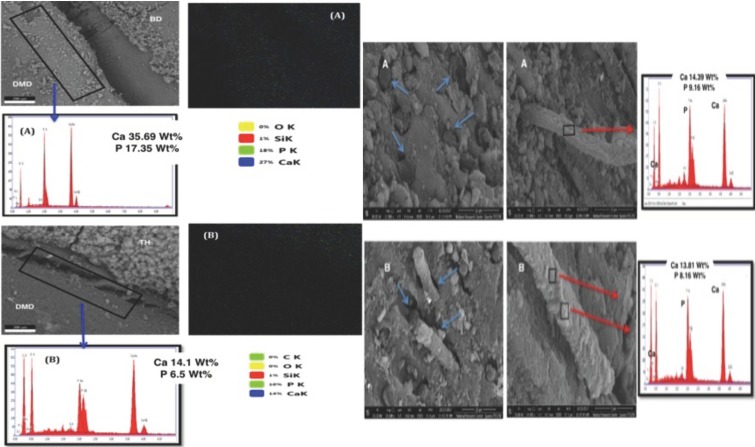
*In the left image*, the blue arrows are pointing to EDS analysis images of DMD adjacent to BD (A) and DMD adjacent to TH (B). They show significantly higher Ca & P Wt% peaks in case of BD. It also shows the surface mapping images of DMD surfaces with higher Ca (27 % as blue rectangle) & P (18 % as light green rectangle) in case of BD with apparent Ca distribution on BD and adjacent DMD surfaces (A) as blue dots. These results were obtained after 6 months of storage. *The right image shows* subsurface layer of the pulpal cavity floor as a transverse section perpendicular to dentinal tubules (A) Beneath Biodentine, and (B) Beneath TheraCal LC, showing, the dentinal tubules filled with rod-like mineralized structure (blue arrows). The red arrows point to EDS images showing that these formed rod like structure after 6 months of storage are formed of Ca & P.

**Figure 3:**
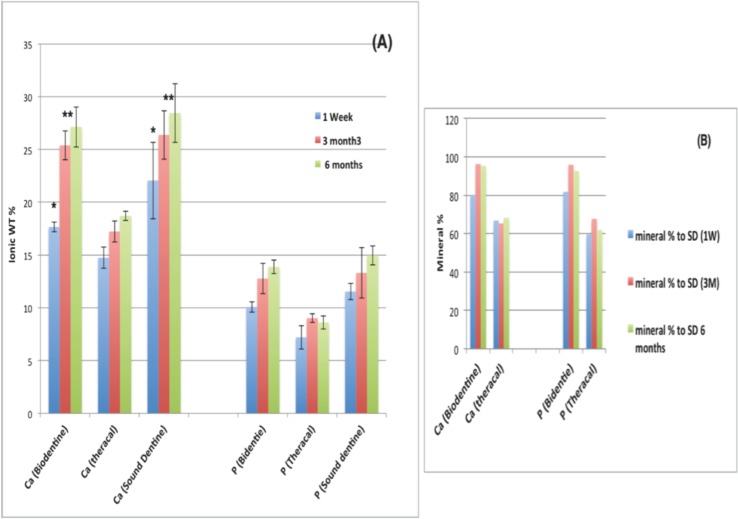
Two bar charts show the Ca and P Wt % in DMD in close contact to both Biodentine and TheraCal after period of 1 week, 3 and 6 months of storage in the PBS (A) ± standard deviations where ** means the highest statistically significant groups and * the second highest means with statistically significant values. The mineral % of Ca and P ions in relation to SD afetr the three storage periods (B).

The pH cycling approach produced a rough porous surface in the targeted areas at the floor of the prepared cavity and at the axial wall surface. The later areas showed lower Ca, P and O (oxygen) content in comparison to the rest of protected dentin surface (Fig. 1). After 6 months of storage, surface mapping and EDS measurements of Biodentine and DMD in close contact to Biodentine showed significantly higher amounts of Ca and P ions than that contacting TheraCal LC. Carbon (C) content was higher for the composite calcium silicate based material (TheraCal LC) (Fig. 2A,B, left image). On the other hand, the subsurface layer beneath both materials showed almost complete filling of all intra-tubular dentin with mineralized tag or rod-shaped structure of similar Ca and P composition (Fig. 2A,B, right image). Two-way ANOVA showed statistically significant differences for Ca and P wt% between DBD adjacent to Biodentine and DMD adjacent to TheraCal LC (*p* < 0.05). It also showed a statistically significant difference between Ca (wt%) values within one week and the other two intervals (3 & 6 months) (*p*-value < 0.05). Comparison of Ca and P wt% as detected from EDS of DMD in close contact to both tested materials is shown in Fig. 3A. The Ca and P wt% of DMD in relation to SD throughout the three tested periods is shown in Fig. 3B.

## Discussion

The remineralization ability of two common and recently released HCSCs, resin-free (Biodentine) and resin-based (TheraCal LC), was estimated in the current study using a cell-independent model. As the reminralization process was identified by increases in Ca and P at the demineralized dentin zone (6), both tested HCSCs have significantly remineralized the DMD region. However, there was a significant increase in Ca and P wt% in DMD adjacent to Biodentine compared with TheraCal LC. This is in accordance with a previous in vitro study that compared such HCSCs with other cement groups like MTA (6). Biodentine, in the current study, generated a significantly higher intensity of remineralizing elements over time throughout all the tested intervals in comparison to TheraCal LC.

The significantly different ability between the two tested HCSCs to release Ca, which is essential for the remineralization process, was previously related to the difference in the released ion detecting method (13,14). However, other issues that are material-related may be more significant. Firstly, the composition of Biodentine capsule powder was reported to have as a major constituent (80.1%) tricalcium silicate (13). Biodentine liquid contains calcium chloride, which acts as a reaction acclerator, hydrosoluble polymer that functions as water reducing agent and water (15). On the other hand, TheraCal LC in the original patent sheet stated that it consists of 45% Portland cement type III, that acts as a Ca ion source, and a resin matrix (43%) (16). Thus it has almost equal amounts of Ca silicate structure and resin matrix, however safety data sheet report that TheraCal LC has less amount of the resin matrix (10–30%) which mainly consists of polyethelyene glycol dimethacrylate, and Portland cement still does not exceed 50 % of the total composition (17). This situation may denote lesser amount of Ca ions source matrix in relation to resin matrix.

Secondly, the setting process for both materials involve hydration chemical setting reactions, resulting in a hydrophilic cement. The hydration process in Biodentine occurs in more efficient aqueous medium found in addition to moisture found in dentin. The reaction results in the formation of calcium silicate hydrate and calcium hydroxide that is produced in large amount (13,18,19). On the other hand, TheraCal LC does not include water for material hydration. It depends on the water uptake from the environment and its diffusion within the material. Therefore, the manufacturer’s instructions require placing the material on moist dentin (13,20). It was reported that TheraCal LC hydration is incomplete because of the limitation of moisture diffusion from the pulp-dentin complex into the set material (13). Hence, the limitation of water medium that facilitates the cement solubility and ion release is more limited in the case of TheraCal LC. In addition, it has been referred to as a light-curable MTA-cement (14). As a result, the setting reaction depends on polymerization of the resin component. That is why it was confused with Portland cements and was excluded, within previous literature, from being classified as a hygroscopic dental cement like; MTA, cements based on bioceramics, calcium silicate, or calcium sulfate (21). The polymerized resin matrix with increased viscosity may also act as a barrier limiting a high rate of ion release. That explanation agrees with previous studies (6,13) that attributed the lower Ca release, and hence lower remineralization efficiency of TheraCal LC than Biodentine and ProRoot MTA, to the different Ca ion release kinetics of each material.

SEM images of the interfacial zone, in the current study, just underneath both materials revealed the formation of tag like structures without significant differences between them. These tags were seen previously with Biodentine and glass ionomer when examined by different microscopy and spectroscopy methods and were attributed to Biodentine’s ability to form calcium hydroxide associated with high pH (12) (9). Furthermore, it released high amount of calcium and silicon ions that stimulate remineralization and create a “mineral infiltration zone” along the dentin-cement interface. Similar tag-like mineral structures infiltration were also observed at the TheraCal LC dentin interface in the present study. Although TheraCal LC does not appear to form calcium hydroxide during setting, it releases Ca ions and produces Ca apatite on its surface (13). All previous *in-vitro* studies had findings related to their associated specific conditions and limitations. Nevertheless, clinical reports found a noteworthy ability of TheraCal LC to from reparative dentin with no significant difference from MTA (22). Biodentine also reported high clinical success in reparative dentin formation in both primary and permanent teeth for indirect pulp capping (23).

Design of the prepared cavity, in the current study, tried to control the remaining dentin thickness and type within deep dentin structure over the pulp. However, the variation of the extent of dentin mineralization and its permeability is still a limitation. Using teeth halves has a distinct advantage in that it tries to improve the standardization process. The artificial carious lesion as induced by the pH cycling approach has been shown to result in artificial caries dentin similar to that of natural caries affected lesions (11). However these artificial lesions lack the complex oral environment and interaction with living pulpal tissue, which is a limitation of the current study. Both resin-free (Biodentine) and resin-based (TheraCal LC) HCSCs may have different interactions with such living cells (24). The later needs further research using more complex models combining artificial carious lesions in the presence of living pulp cells.

## Conclusions

Based on the results and within the limitations of the present *in vitro* study, Biodentine is able to enrich the adjacent dentin area with significantly higher amounts of Ca ions that are essential for the remineralization process. Both tested HCSCs showed significant interaction and formation of mineralized tag-like structure within the artificial DMD interface area.
